# Event Monitoring and Evaluation by Community Pharmacists in Japan: A Pilot Study on Fenofibrate and Pemafibrate

**DOI:** 10.2174/1574886317666220224142511

**Published:** 2022-07-21

**Authors:** Masao Takahashi, Nobuhiro Ooba, Marina Nagamura, Makoto Ushida, Eiji Kawakami, Masaomi Kimura, Tsugumichi Sato, Yoshinori Takahashi, Junichi Tokuyoshi, Hajime Hashiba, Miwako Kamei, Choichiro Miyazaki, Mitsuaki Shimada

**Affiliations:** 1 DEM Committee, Japan Pharmaceutical Association, Tokyo, Japan;; 2 Department of Clinical Pharmacy, Nihon University School of Pharmacy, Chiba, Japan;; 3 Faculty of Pharmacy, Meijo University, Aichi, Japan;; 4 Department of Computer Science and Engineering, Shibaura Institute of Technology, Tokyo, Japan;; 5 Department of Pharmacy, Faculty of Pharmaceutical Sciences, Tokyo University of Science, Chiba, Japan;; 6 Faculty of Pharmaceutical Sciences, Teikyo Heisei University, Tokyo, Japan

**Keywords:** Retrospective cohort study, new-user design, event monitoring, concurrent control, primary data collection, community pharmacist

## Abstract

***Background*:** The Japan Pharmaceutical Association has conducted drug event monitoring to detect drug events related to pemafibrate. As there are a few studies on the safety of pemafibrate in clinical settings, a pilot study evaluating the association between drug use and detected events was performed in Japan.

***Aims*:** In this study, the association between detected events and the use of pemafibrate, utilizing pharmacy records maintained by community pharmacists, was investigated. We identified the new-user cohort using a test and active comparison drug and collected the baseline information. An active comparison group comprising new users was used to assess the events.

***Methods*:** A retrospective cohort study using questionnaires regarding baseline and event data was conducted by community pharmacists belonging to the Japan Pharmaceutical Association. The incidence of event and estimated hazard ratio were calculated using the Cox proportional hazards model that was adjusted for confounding factors, such as age and sex.

***Results*:** A total of 1294 patients using pemafibrate and 508 patients using fenofibrate were identified as new drug users. The most reported events involving suspected adverse reactions and add-on drugs were increased blood pressure and lipid-lowering effects with pemafibrate use, and nasopharyngitis, pruritus, dizziness, and lipid-lowering effects with fenofibrate use. No significant differences were found in commonly occurring events, except that an add-on anti-hypertensive drug has been used by pemafibrate users compared to fenofibrate users.

***Conclusion*:** This study conducted by pharmacists can facilitate the safety assessment of newly marketed drugs, as few drug use investigations with a comparator are carried out by the Japanese authority for pharmaceutical companies. However, further research is required.

## 1 INTRODUCTION

Information regarding a medicine’s safety is generally limited during the early post-marketing period, even though the approved medicine begins to be widely used in clinical practice. To provide complementary information on drug safety, prescription-event monitoring (PEM) [1] was developed in the UK and, over time, evolved into modified PEM (M-PEM) [2, 3]. Studies using similar methodologies have been conducted in New Zealand and Japan [2, 4]. PEM in Japan (J-PEM) [5, 6], which was launched in 1997, has been suspended in 2004. Instead, another investigation of drug-related events, called drug event monitoring (DEM), has been implemented in 2002 by community pharmacists under the purview of the Japan Pharmaceutical Association (JPA) [7] to monitor events occurring in prevalent users of some pre-selected study drugs [8]. In real-world clinical practice, studies using primary data obtained through event monitoring may help generate study hypotheses, detect the safety signals of drugs, and promote spontaneous reporting systems, even though the limitations for hypothesis testing on safety concerns regarding drugs in the early phases after marketing are well known [9]. Collecting baseline information and information on the design of M-PEM [2] and setting a concurrent comparison group may help overcome some of the limitations of the conventional method of current DEM.

In a risk management plan (RMP) for newly marketed drugs, severe or frequent adverse drug reactions are summarized as important specified risks. Although RMP [10] was introduced in 2013 in Japan, a comparison group is required to evaluate whether the risk of known or unknown detected events is associated with the use of a drug. We can expand the event monitoring role of DEM by incorporating a component of retrospective cohort studies using the new-user design in the schema of DEM.

**Fig. (1):**
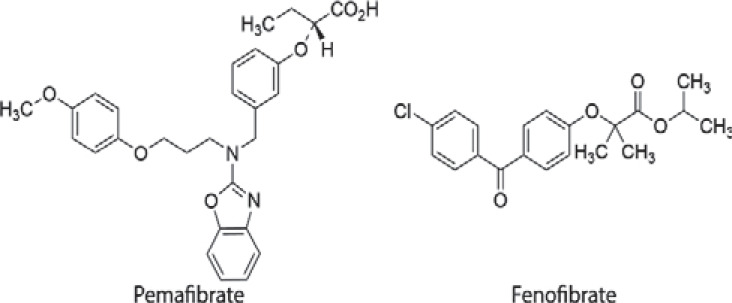
Chemical structures of pemafibrate and fenofibrate.

Newly marketed drugs with indications for chronic diseases, such as hypertriglyceridemia, dispensed in many pharmacies would be suitable as study drugs for DEM. Fibrates were recommended by therapeutic guidelines as they have good efficacy in lowering fasting triglyceride levels [11, 12]. Fenofibrate as a treatment has been marketed in some countries, including the United Kingdom and the United States, whereas pemafibrate, with the profile of a novel selective peroxisome proliferator-activated receptor α modulator (SPPARMα) [13], is approved only in Japan as of May 2021 (Fig. **1**). A phase III randomized controlled trial for pemafibrate suggested a favorable benefit-risk balance in comparison to fenofibrate [14]. In addition, systematic reviews of pemafibrate reported no significant difference in total adverse events between pemafibrate and placebo groups [15]. However, elevated low-density lipoprotein cholesterol (LDL) was indicated as a potential risk in the RMP for pemafibrate [16]. Safety information needs to be complemented by observational studies, as randomized trials may not accurately represent patients in usual clinical practice [17].

### 1.1 Aim of the Study

This study aimed to detect a drug-related event and evaluate the association between the event and drug use using a retrospective cohort study for pemafibrate as a newly post-marketed drug and fenofibrate as an active comparison drug in the new schema of DEM in Japan.

## 2 MATERIALS AND METHODS

A retrospective cohort study using a new-user design was conducted in February 2020 at 5562 of the 48382 community pharmacies belonging to the JPA [18]. The JPA sent questionnaires to all the community pharmacies, where pharmacists completed the forms using paper-based or electronic health records, including the patients’ preferences, allergies, prescriptions, and history of diseases. Except for pharmacies with no study patients or pharmacies that refused to participate in the study, pharmacists from 2343 community pharmacies completed the questionnaires. They were anonymized using a study ID for each pharmacy into the web-based form that was prepared in advance. The questionnaire included baseline data (demographic, co-medication, and comorbidity) within six months before the starting date of the use of the study drug and drug and events data during the follow-up period between September and December 2019.

Study patients were restricted to new users of the study drug, namely patients who were users of the pharmacy from before February 2019 and had newly started taking the study drug in September 2019 after six months of non-study drug use. The observation period was from September 1^st^ to December 31^st^, 2019, and follow-up was conducted four months after the initiation of the drug. Since patients can freely access any community pharmacy or medical institution without referral in Japan, we limited the study population to those who regularly used the same pharmacy by referring to visit records spanning more than seven months before the start of the drug use to identify new users of the drug and prevent the loss of participants before follow-up [19]. Patients who had only one visit on the start date of the prescription, including new users, were excluded because they could not be followed up.

### 2.1 Exposure

Similar to J-PEM, our study design set a concurrent control [20]. To have similar indications for both test and active comparator drugs, we selected a pair comprising a test drug (pemafibrate) and a comparator drug (fenofibrate). The drugs used as study targets had all been marketed for more than one year because the supply of newly marketed drugs is generally limited to 14 days to one year from the first day of the month as per the National Health Insurance Drug price listing in Japan [21].

Since determining which study drug (test or comparator) caused the event is difficult, we did not include patients who switched from the comparator to the test drug or from the test drug to the comparator drug. In addition, we excluded patients who had previously used any of the study drugs in each drug pair to prevent the effect of bias [22], owing to many test drug users having previously used the comparator drug.

### 2.2 Data Collection of Events

We collected events data, including the incidence date of the event from the health record of the pharmacy, wherein an event was defined as any suspected drug reaction; unexpected deterioration (or improvement) in a concurrent illness; the reason for stopping the use of the study drug (if stopped) or adding a new drug (if added). According to Article 25-2 of the Pharmacists Act in Japan, the pharmacist provides the patient or a person caring for the patient the necessary guidance based on pharmaceutical knowledge and monitors the effects of the drugs and changes in physical condition during drug use. Information on add-on drugs may be used to characterize the event. Adverse events were partly defined using the definition of M-PEM [2]. Although our basic study design refers to M-PEM, the process of data collection and definition of an event was slightly different. The questionnaires for M-PEM were sent to prescribers, while community pharmacists collected the necessary data from the health records of the pharmacies included in our study. Therefore, the definition of an event could not include the diagnosis by a medical doctor. However, we collected complementary information regarding the judgement of causality by pharmacists for the reported event, such as “unlikely,” “probable,” and “not assessable” [23].

The Medical Dictionary for Regulatory Activities Terminology (MedDRA) version 22.1 was used to encode the reported event; we selected the lowest level terms in the MedDRA [24], which were converted to the preferred term level and counted as an event.

### 2.3 Statistical Analysis

We described summary statistics for demographic characteristics, current smoking, alcohol consumption, taking over-the-counter drugs, comorbidities (dyslipidemia, hypertension, diabetes mellitus, myocardial infarction, and cancer), co-medications (anti-hypertensives, lipid-lowering drugs, anti-diabetes drugs, anti-ulceratives, steroids [except those for external use], laxatives and anti-depressants/hyp-notic drugs), and history of hospital admission at a six-month baseline period. A standardized difference was calculated between paired drugs and was considered meaningful when greater than 0.1 [25].

We calculated the incidence proportions of the events in patients during treatment with test or comparator drugs and estimated the risk ratio and its 95% confidence interval (CI). If the same event was reported more than once, only the first event was considered. For some events, time-to-event occurrence was depicted using a Kaplan–Meier curve and compared using a log-rank test. The Cox proportional hazards model was used to estimate the unadjusted and adjusted hazard ratio (HR) of the test drug on the event compared to the comparator drug. To adjust the HR, we used covariates with a standardized difference of 0.1 or more. In this analysis, we defined the observation period for assessing the event as follows: the period from the start date of the study drug to the incidence date, date of switching or stopping the study drug, last visit date, or the end of the observation period date, *i.e*., December 31^st^, 2019, whichever came first.

Results with a p-value <0.05 were considered statistically significant. All analyses were conducted using SAS version 9.4 (SAS Institute Inc., Cary, NC, USA).

## 3 RESULTS

Commonly dispensed drugs in the study period were pemafibrate (n=5020) as the test drug and fenofibrate (n=2825) as the comparator drug. Of these, 1294 patients using pemafibrate (26%) and 508 patients using fenofibrate (18%) were identified as new users of the drugs. Table **1** shows the data on the baseline characteristics of the study patients. Standardized differences in age, smoking, myocardial infarction, lipid-lowering drugs, and history of hospitalization were greater than 0.1.

The proportion of patients who discontinued the drug was 3.9% (n=50) for pemafibrate and 6.3% (n=32) for fenofibrate. Discontinuation because of any suspected adverse drug reaction was 1.5% for pemafibrate and 2.0% for fenofibrate, while the proportion of improvement was 1.4% for pemafibrate and 2.8% for fenofibrate.

In pemafibrate, the incidence proportion (95% CI) of any event was 11.8% (10.2% to 13.7%), while that for fenofibrate was 8.3% (6.2% to 11.0%). The crude risk ratio of any event was higher for pemafibrate users than for fenofibrate users [1.43 (1.03 to 1.98)]. Tables **2** and **3** show the five most reported events and incidence proportions of the drug. For pemafibrate, increased blood pressure (1.3%), myalgia (0.6%), and pain in the extremities (0.5%) were reported. Nasopharyngitis (0.6%), pruritus (0.6%), and dizziness (0.6%) were reported for fenofibrate, and the reported number of cases (incidence proportion) of increased blood pressure was 2 (0.4%).

We have presented the unadjusted and adjusted HR for the common events, including the drug added after the initiation of the test or comparator drug, compared to the fenofibrate as reference (Table **4**). No significant differences were found in the events except for the add-on of an anti-hypertensive (adjusted HR=3.20; 95% CI, 1.36–7.52).

**Fig. (2):**
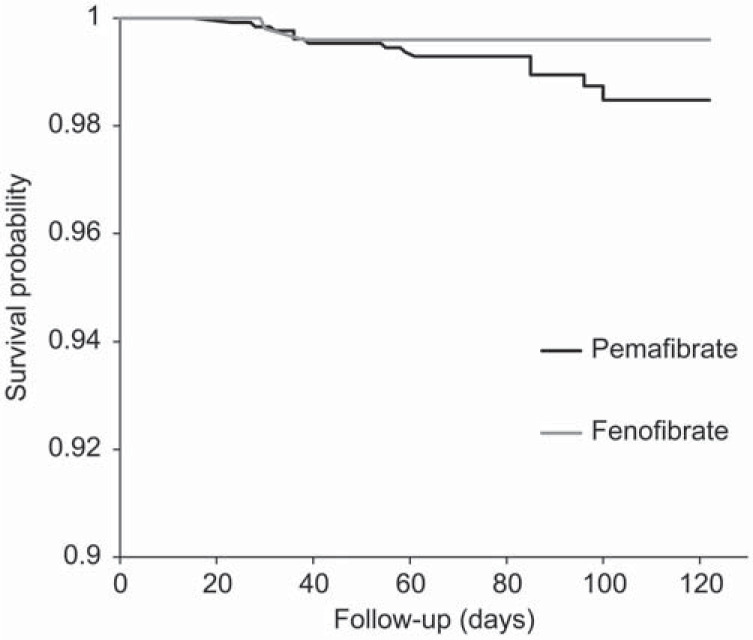
Probability of blood pressure increase among users of pemafibrate and fenofibrate. Survival probability at a time point is the unadjusted probability of not developing blood pressure increase by that time point.

To examine the event of increased blood pressure with the use of pemafibrate, we used the Kaplan-Meier curves for the event during the study period (Fig. **2**). A log-rank test revealed that the time to the event did not significantly differ between the pemafibrate and fenofibrate groups (p=0.08). Furthermore, of the 17 events of increased blood pressure reported for pemafibrate, eight events were determined by pharmacists to be unlikely due to the use of pemafibrate.

## 4 DISCUSSION

The DEM design in Japan could play the dual role of event monitoring and evaluating the detected events [26]. Generally, event monitoring helps generate test hypotheses and strengthen the safety signal of drugs [3]. Groups must be compared in terms of the risk of events to assess the association between newly marketed drugs and events. We identified new users of test or comparator drugs using electronic dispensing data maintained by each pharmacy and followed the study cohort for at least three months. In addition, community pharmacists were able to collect information regarding the event using pharmacy records, demographic data, including over-the-counter drugs for outpatients, and health information about the patients. This process may help evaluate detected signals in the real-world post-marketing of drugs. In the Revision of Good Post-Marketing Study Practice Ordinance implemented in Japan as of April 2018 [27], post-marketing database surveillance and drug use investigation with a comparator group using primary data were newly added to the conventional post-marketing surveillance required by the Japanese authority for pharmaceutical companies. However, in the fiscal year 2018, no drug use investigations were conducted with comparator groups as an additional pharmacovigilance plan of the RMP [28]. DEM by community pharmacists can serve as an option for post-approval safety studies.

The incidence of LDL increase mentioned as an important potential risk in RMP [16] for pemafibrate was small (n=3, 0.2%), while no event was reported for fenofibrate. However, lipid-lowering drug was additionally used 1.9 times more by pemafibrate users than by fenofibrate users; however, this difference is not statistically significant. Therefore, monitoring of lipid levels and recommendations for lifestyle changes may be needed for patients during pemafibrate use.

Gremmels *et al.* suggest that studies on fibrates show inconsistent results related to blood pressure [29]. In addition, some randomized controlled studies did not mention the adverse events on blood pressure [14, 30]. However, in our study, the use of pemafibrate was not related to the risk for events of increased blood pressure, while the add-on of an anti-hypertensive increased significantly in pemafibrate users compared to users of fenofibrate (Table **4**). Although multivariate-adjusted HR for both events of pemafibrate was approximately 3.0, the 95% CI was wide and inconclusive. Our findings for increased blood pressure need to be further replicated in larger sample sizes.

Details of the mechanism for hypertension in pemafibrate are unknown. Gilbert *et al*. suggest that the anti-hypertensive effect of fenofibrate as a comparator drug, such as that observed in our study, results from an action downstream from PPARα activation [31]. In contrast, Yamashita *et al*. reported that pemafibrate showed high selectivity for PPARα compared to conventional fibrates [32]. In our findings, the incidence of increased blood pressure in pemafibrate users was not significant; however, the add-on of anti-hypertensive for pemafibrate may characterize the effect on blood pressure. If the anti-hypertensive effect of fenofibrate is related to PPARα activation, the increase in blood pressure by pemafibrate, which is highly selective for PPARα, may be inconsistent.

This study has several strengths. First, in our retrospective cohort study using primary data from pharmacies, we used a concurrent comparator group comprising new drug users. Therefore, we could estimate the measure of effect, in addition to the identification or confirmation of drug safety signals. Second, we could collect information on comorbidity, co-medication, and lifestyle factors for current smoking, alcohol consumption, and over-the-counter drugs. This is useful as these factors can be adjusted to strengthen or refine the detected signals. Third, the generalizability of our findings is high. As of March 2019, the number of pharmacies in Japan was 59613 [33]. As of February 2020, 48382 pharmacies belonged to JPA, 11.5% of which participated in our study.

Nevertheless, this study has some limitations. First, as our sample size was small, the estimates of the association between drug and events were unstable, and their CIs were wide. For events that were small in number, the Cox proportional hazards model may lack the power to detect the differences between drug groups due to the non-proportional hazard assumption. Second, the follow-up period in our study was relatively short (approximately four months). The rare events that may occur in the long term may have been missed, although the events that may be likely to occur early after the initiation of the drug were captured. Third, the events related to laboratory test values, such as the elevation of creatine kinase, are underestimated. At pharmacies, laboratory test values can only be confirmed by some patients, although some clinics and hospitals attach data of laboratory test values to their prescriptions.

## CONCLUSION

A retrospective cohort study involving the framework of DEM with a comparison group comprising new users of drugs was conducted by community pharmacists in Japan. However, further study is needed as the sample size and the number of events were small for both pemafibrate and fenofibrate users. DEM may help monitor and evaluate safety signals for newly marketed drugs in real-world clinical settings. This design may apply to drug use investigations with a comparison group in pharmacovigilance plans as an RMP by pharmaceutical companies.

## Figures and Tables

**Table 1:** Baseline characteristics of new pemafibrate or fenofibrate users.

-	**Pemafibrate** **(*n* = 1,294)**	**Fenofibrate** **(*n* = 508)**	**Standardized Difference**
Median age (years, IQR)	64 (53−72)	67 (56−76)	–0.240
Male (%)	835 (64.5)	317 (62.4)	0.044
Mean daily dose (mg)	0.3	99.2	-
Number of missing doses	7	0	-
Mean observation period (days)	99.5	101	-
Current smoker (%)	262 (20.2)	81 (15.9)	0.112
Current drinker (%)	503 (38.9)	190 (37.4)	0.030
Comorbidity (%)	-	-	-
Dyslipidemia	849 (65.6)	332 (65.4)	0.005
Hypertension	798 (61.7)	332 (65.4)	-0.077
Diabetes mellitus	440 (34.0)	151 (29.7)	0.092
Myocardial infarction	29 (2.2)	3 (0.6)	0.140
Cancer	33 (2.6)	10 (2.0)	0.039
Co-medication (%)	-	-	-
Anti-hypertensives	824 (63.7)	340 (66.9)	-0.068
Lipid-lowering drugs except fibrates	525 (40.6)	170 (33.5)	0.148
Anti-diabetes drugs	449 (34.7)	156 (30.7)	0.085
Anti-ulceratives	286 (22.1)	96 (18.9)	0.079
Steroids except those for external use	16 (1.2)	4 (0.8)	0.045
Laxatives	104 (8.0)	39 (7.7)	0.0113
Anti-depressants/ hypnotic drugs	192 (14.8)	71 (14.0)	0.025
History of admission (%)	8 (0.6)	0 (0)	0.112

Abbreviation: IQR: interquartile range.

**Table 2:** The five most reported events in pemafibrate users.

**Event**	**Number (%) of Events in Pemafibrate Users (n=1,294)**
Adverse reaction	-
Increased blood pressure	17 (1.3)
Myalgia	8 (0.6)
Pain in extremity	7 (0.5)
Pruritus	5 (0.4)
Abdominal discomfort	5 (0.4)
Add-on drug	-
Lipid-lowering drug	48 (3.7)
Anti-hypertensive	47 (3.6)
Anti-diabetes drug	35 (2.7)
Non-steroid anti-inflammatory drug	13 (1.0)
Anti-ulcerative	12 (0.9)

**Table 3:** The five most reported events in fenofibrate users.

**Event**	**Number (%) of Events in Fenofibrate Users (n=508)**
Adverse reaction	-
Nasopharyngitis	3 (0.6)
Pruritus	3 (0.6)
Dizziness	3 (0.6)
Increased blood pressure	2 (0.4)
Myalgia	2 (0.4)
Renal impairment	2 (0.4)
Cough	2 (0.4)
Hypoesthesia	2 (0.4)
Abnormal hepatic function	2 (0.4)
Add-on drug	-
Lipid-lowering drug	10 (2.0)
Anti-diabetes drug	6 (1.2)
Anti-hypertensive	6 (1.2)
Anti-ulcerative	4 (0.8)
Laxative	3 (0.6)

**Table 4:** Unadjusted and adjusted hazard ratios for the events reported most in both pemafibrate and fenofibrate users.

**Event**	**Unadjusted HR (95% CI)**		Adjusted* HR (95% CI)	
	**Fenofibrate**	**Pemafibrate**	**Fenofibrate**	**Pemafibrate**
Adverse reaction	-	-	-	-
Increased blood pressure	1.0	3.39 (0.78–14.68)	1.0	3.40 (0.77–14.93)
Pruritus	1.0	0.66 (0.16–2.74)	1.0	0.77 (0.18–3.29)
Dizziness	1.0	0.52 (0.12–2.34)	1.0	0.46 (0.09–2.01)
Add-on drug	-	-	-	-
Lipid-lowering drug	1.0	1.93 (0.97–3.81)	1.0	1.87 (0.94–3.71)
Anti-hypertensive	1.0	3.15 (1.35–7.37)	1.0	3.20 (1.36–7.52)
Anti-diabetes drug	1.0	2.33 (0.98–5.54)	1.0	2.11 (0.88–5.05)
Anti-ulcerative	1.0	1.19 (0.38–3.68)	1.0	1.10 (0.35–3.51)
Laxative	1.0	0.99 (0.19–5.08)	1.0	1.03 (0.18–5.70)

*Adjusted for age, sex, current smoking, lipid-lowering drug, and history of admission.

Abbreviations: HR, hazard ratio; CI, confidence interval.

## Data Availability

The authors confirm that the data supporting the findings of this research are available within the article.

## STANDARDS OF REPORTING

STROBE guidelines were followed in this study.
